# 16S rRNA Gene Amplicon Sequencing Data from Flooded Rice Paddy Mesocosms Treated with Different Silicon-Rich Soil Amendments

**DOI:** 10.1128/MRA.00178-21

**Published:** 2021-07-08

**Authors:** Gretchen E. Dykes, Clara S. Chan, Angelia L. Seyfferth

**Affiliations:** a Department of Plant and Soil Sciences, University of Delaware, Newark, Delaware, USA; b Department of Earth Sciences, University of Delaware, Newark, Delaware, USA; c Delaware Biotechnology Institute, University of Delaware, Newark, Delaware, USA; Loyola University Chicago

## Abstract

How silicon-rich soil amendments impact the microbial community is unresolved. We report 16S rRNA gene sequencing data from flooded rice paddy mesocosms treated with different silicon amendments sampled over the growing season. We generated 11,678 operational taxonomic units (OTUs) and found that microbial communities were significantly different across treatments, time points, and biospheres.

## ANNOUNCEMENT

Silicon (Si)-rich soil amendments can decrease inorganic As in rice (1–7), but how they impact the microbial community is unresolved. These amendments can release organic carbon, nutrients, and/or electron acceptors, with potential impacts on microbially driven biogeochemical cycles.

We sequenced 16S rRNA genes from samples collected from three biospheres (root plaque, rhizosphere, and bulk soil) over the rice life cycle from unamended paddy mesocosms and those amended with rice husk, husk char, or calcium silicate (*n* = 3 per treatment) at the University of Delaware RICE facility in Newark, DE (1). Paddy soils were amended in year 1 and then cultivated with rice for two growing seasons; the soil samples described here were collected in year 2 (8). Of the 49 rice (Oryza sativa L. cv. Jefferson) seedlings planted in each mesocosm (8), 5 random plants were contained in soil-filled 100-μm nylon mesh bags to define the rhizosphere (Table 1). At each time point, one bag was pulled per paddy, bulk soil was collected from surrounding soil, and the bagged plants were placed in ethanol-sterilized containers for immediate transport to the lab. Roots were separated from the root mass with sterilized instruments and cleaned of rhizosphere soil by vortexing twice in 25 ml of sterile water (18 MΩ · cm). Bulk soil, rhizosphere soil, and cleaned roots were frozen at −20°C prior to DNA extraction. For plaque sampling, cleaned roots were thawed, sonicated twice for 30 s in 10 ml of phosphate buffer solution, and centrifuged at 18 × *g* for 5 min (modified from reference 9), and DNA was immediately extracted from the pellet.

**TABLE 1:** Sampling time points and corresponding bag sizes

Time point (days after transplant)	Rice growth stage	Bag size (diam by length [cm])
20	Vegetative	10.16 by 20.3
42	Early reproduction	12.7 by 20.3
71	Heading	15.2 by 31.8
88	Grain ripening	15.2 by 31.8
98	Grain maturity	15.2 by 31.8

DNA was extracted with the DNeasy PowerSoil DNA extraction kit (Qiagen) with modifications. Initially, 200 μl of PowerBead solution was replaced with 200 μl of phenol-chloroform-isoamyl alcohol (25:24:1) at neutral pH. The protocol was followed according to manufacturer’s instructions until column binding, when equal parts lysate, solution C4, and 100% ethanol were homogenized and then loaded onto the DNA binding column. Next, the column was washed with 100% ethanol (650 μl) and then solution C5 (500 μl). Finally, DNA was eluted with molecular biology-grade water.

The V4-V5 16S rRNA gene region was amplified using primers 515F-Y and 926R (10) at the Joint Genome Institute (JGI), according to the JGI “iTag Sample Preparation for Illumina Sequencing” protocol, available at https://jgi.doe.gov/wp-content/uploads/2019/07/iTag-Sample-Preparation-for-Illumina-Sequencing-SOP-v1.0.pdf. Samples were sequenced with paired-end 2 × 300-bp Illumina MiSeq reads. Sequences were demultiplexed, quality filtered, clustered at 97% similarity, and checked for chimeras with the JGI iTagger pipeline (11). To filter out the rarest operational taxonomic units (OTUs), we used QIIME 2 (12) to remove OTUs that only occurred in one sample and OTUs that had fewer than 10 assigned reads across the entire data set. We assigned taxonomy using the sklearn naive Bayes feature classifier in QIIME 2, trained with the SILVA database version 132. R version 3.6.2 (13) was used for statistical analyses, and default parameters were used unless otherwise noted.

**FIG 1:**
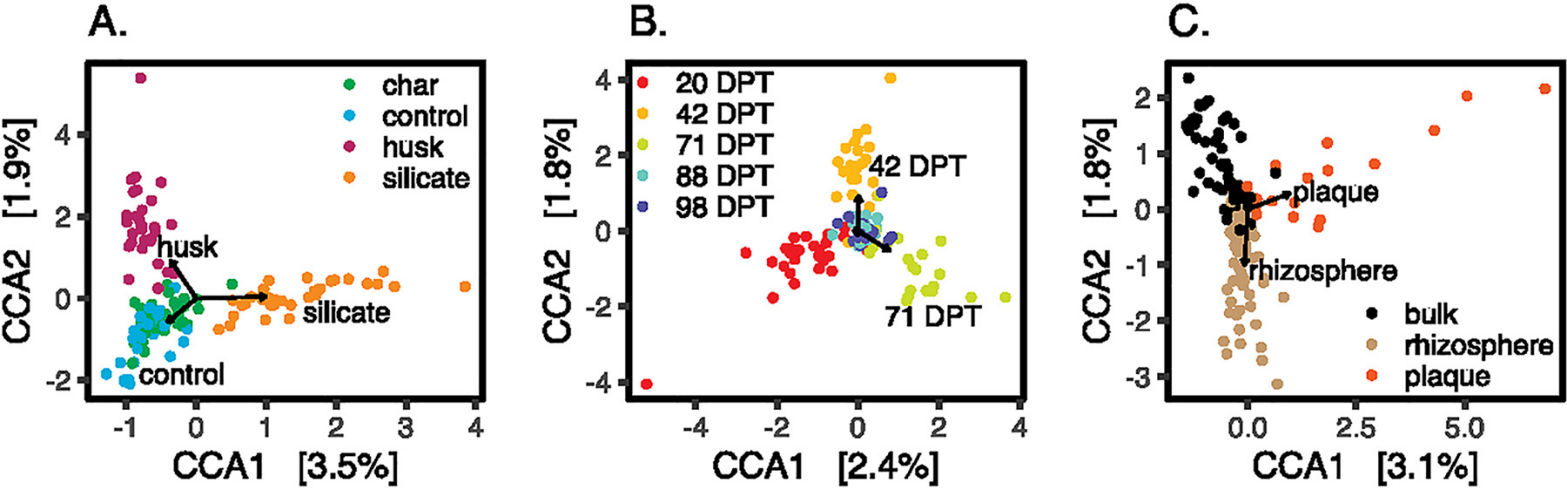
Constrained correspondence analysis of soil samples constrained by treatment (A) (ADONIS; *R*^2^ = 0.09 and *P* = 0.001), time point (B) (ADONIS; *R*^2^ = 0.11 and *P* = 0.001), or biosphere (C) (ADONIS; *R*^2^ = 0.05 and *P* = 0.001). DNA was obtained from fewer plaque samples than rhizosphere or bulk soil. Ordination plots were generated in R (13) with the package phyloseq (15). DPT, days since transplantation.

We obtained 69,402,988 raw reads from 130 samples with high-quality DNA extraction, 37,072,594 (53%) of which remained after quality filtering. By clustering at 97% identity, we obtained 11,678 OTUs, only 3,914 of which were assigned taxonomy at the genus level. *Bacteroidetes* and *Proteobacteria* were the dominant phyla in these samples. The community composition was significantly different between Si treatments, time points, and biospheres (Fig. 1).

### Data availability.

The raw 16S rRNA gene sequencing data from this project have been deposited in the NCBI BioProject SRA database (14) under accession number PRJNA690162, with samples described here from the 2016 growing season.
